# Individual Perceptions and Self-Care Practices for Abdominal Pain: A Cross-Sectional Survey Conducted in Italian Community Pharmacies

**DOI:** 10.3390/pharmacy14060133

**Published:** 2026-09-14

**Authors:** Corrado Giua, Nicolina Paola Floris, Lisa Boschetti, Lorenzo Turati, SGCP, Enrico Keber

**Affiliations:** Società Italiana Farmacia Clinica (SIFAC), Viale Regina Margherita 30, 09124 Cagliari, Italy; corrado.giua@gmail.com (C.G.); ni.26@hotmail.it (N.P.F.); lisaboschetti91@gmail.com (L.B.); dr.lorenzoturati@gmail.com (L.T.); segreteria@sifac.it (SGCP)

**Keywords:** abdominal pain, community pharmacy, self-care, self-medication, pharmacist counselling, cross-sectional survey, SIFAC, Italy

## Abstract

Background: Abdominal pain is one of the most common complaints in community pharmacy, yet how individuals define it, which symptoms they associate with it, and how they manage it through self-care remains poorly characterized. Objective: This study aimed to investigate individual perceptions, definitions, and self-care behaviors related to abdominal pain among adults attending Italian community pharmacies and to explore healthcare-seeking behaviors and needs. Methods: A cross-sectional observational survey was conducted among adults attending community pharmacies across Italy. Eligible participants were aged ≥18 years and provided consent for data processing; individuals with cognitive or language barriers were excluded. Thirty-three SIFAC clinical pharmacists administered an expert-reviewed 18-item digital questionnaire (October–December 2025), covering pain perception, healthcare needs, and self-care behaviors. Data were analyzed descriptively. Results: 406 pharmacy customers were enrolled (66.7% female; mean age 44.3 years). Over 93% reported at least one episode in the previous 12 months. The term was applied heterogeneously across localizations, symptom profiles, and attributed causes. The pharmacist was the preferred professional for mild-to-moderate episodes (29.9%) and high-intensity episodes without alarm signs (40.2%). Conversely, the physician was most frequently consulted for high-intensity episodes with alarm signs (53.4%) and first-time episodes (16.1%). Self-medication was reported by 89.4%, with probiotics and intestinal flora products (57.4%), antispasmodics (43.8%), and antacids and gastric acid suppressants (32.5%) most commonly used. Despite this, 55.3% expressed insecurity about autonomous management. Conclusions: The findings indicate that community pharmacists represent an important point of contact in the initial management of abdominal pain among pharmacy customers. The heterogeneous symptom profiles, widespread use of self-medication, and uncertainty reported by many respondents about autonomous management support the potential value of structured symptom assessment, personalized counselling, and clear guidance on when medical evaluation is warranted.

## 1. Introduction

Abdominal pain is among the most prevalent gastrointestinal complaints in the general population, with functional disorders of this type affecting a substantial proportion of adults worldwide and carrying a significant burden on health-related quality of life [1,2,3]. Although often perceived as a self-limiting condition, abdominal pain may arise from a wide spectrum of underlying causes, ranging from functional gastrointestinal disorders, food intolerances, and intestinal infections to gynecological pathology and psychosomatic manifestations, with substantial variability in how individuals perceive and describe it [4]. This clinical heterogeneity makes the distinction between situations potentially amenable to self-care and those warranting professional medical assessment particularly important. For instance, while mild and transient gastrointestinal symptoms are generally manageable through self-care or a community pharmacist, the presence of specific alarm signs, such as recent unexplained weight loss, blood in the stools, or an abnormal abdominal mass, represents a clear red flag that requires professional medical referral [5,6].

To properly investigate these behavioral patterns, a clear distinction between health-seeking concepts is essential. Consistent with the Global Self-Care Federation (GSCF) [7], self-care encompasses a broad range of behaviors, including self-recognition and self-monitoring of symptoms, self-management of health conditions, and responsible use of medicines, either independently or in partnership with healthcare professionals. Within this broader framework, self-medication is defined by the World Health Organization (WHO) [8] as “the practice whereby individuals treat their ailments and conditions with medicines which are available and approved without prescription and which are safe and effective when used as directed”.

Community pharmacies are frequently the first port of call for people seeking initial management of abdominal complaints, with gastrointestinal symptoms among the leading reasons for patient-initiated consultations [9,10], and the pharmacist is identified as one of the most accessible and trusted healthcare professionals for this type of presentation [11].

In Italy, community pharmacies are formally integrated into the National Health Service (NHS) [12], and the Italian Society of Clinical Pharmacy (SIFAC) has actively promoted evidence-based pharmacy practice and practice-based research since its foundation in 2012 [13].

Cross-sectional surveys conducted in real pharmacy settings have proved especially valuable in community pharmacy research, offering a direct window into patient experiences and behaviors that are otherwise difficult to capture through experimental or simulated designs. In the field of gastrointestinal complaints, a survey-based study across Italian community pharmacies mapped the therapeutic history of 1020 individuals with upper gastrointestinal symptoms and generated practice-relevant evidence for pharmacist counselling. Notably, 38.1% of patients did not seek medical advice, and 42.0% relied on non-prescription therapies [14]. Similar approaches have been used to assess the quality of pharmacist–patient communication [15], with a study involving 460 community pharmacy users identifying significant gaps between patient expectations and perceptions across all assessed domains. Studies have also characterized gaps in pharmacist history-taking and triage in simulated-patient settings [16,17]. Only 2.3% of pharmacists correctly identified a simulated gastric ulcer presentation [16], while poor counselling and dispensing practices were observed in 81.3% and 67.3% of pharmacies, respectively, in response to a gastroesophageal reflux complaint [17]. Research has also explored how misaligned expectations between patients and pharmacists affect the quality of advice provided [18]. When rigorously designed and conducted, surveys of this kind can generate evidence that is directly actionable in clinical pharmacy practice.

Yet, for all the methodological progress in pharmacy-based survey research, the subjective experience of abdominal pain at the pharmacy counter has remained largely uncharted. Previous Italian studies have focused on musculoskeletal pain [19] and upper gastrointestinal symptoms [14], while international research has highlighted persistent gaps in pharmacist responses to abdominal and gastric complaints [16,17]. A systematic account of how people define, experience, and self-manage abdominal pain in this setting is still missing.

The present study was designed to address this gap. Specifically, it aimed to (a) characterize consumer definitions and perceptions of abdominal pain in terms of localization, associated symptoms, intensity, and attributed causes; (b) explore healthcare-seeking behaviors and needs, including the situations in which individuals seek advice from a pharmacist or consult a physician; (c) describe self-care behaviors, including product categories used, information channels consulted, and perceived confidence in autonomous management; and (d) identify opportunities for improving pharmacist counselling, patient education, and triage within the community pharmacy setting.

## 2. Materials and Methods

### 2.1. Study Design

A cross-sectional observational survey was conducted among adults (18 years or older) attending community pharmacies in Italy. The survey instrument was developed by the SIFAC Research Centre in September 2025 and reviewed by a multidisciplinary scientific board comprising a gastroenterologist, a general practitioner, and a pediatrician, who assessed its clinical appropriateness and scientific relevance. The final 18-item expert-reviewed questionnaire was structured into four sections: Section A (sociodemographic characteristics); Section B (perception and definition of abdominal pain: episode frequency, localization, associated symptoms, perceived intensity, and attributed causes); Section C (healthcare needs, clinical situation driving patients to contact and consult a pharmacist or a physician, importance of pharmacy services, and additional support requested); and Section D (self-care behaviors, product categories used, information channels consulted, and self-management confidence). Response formats included single-choice and multiple-choice items (maximum two to three selections per item). Service importance in Section C was measured on a four-point scale ranging from 1 (not at all important) to 4 (very important). Self-management confidence in Section D was assessed on a four-point scale ranging from 1 (not at all confident) to 4 (completely confident). The full questionnaire is provided as Supplementary Table S1.

This survey is reported in accordance with the Strengthening the Reporting of Observational Studies in Epidemiology (STROBE) statement for cross-sectional studies [20]. The completed STROBE checklist is provided in the Supplementary Materials.

### 2.2. Participant Recruitment and Data Collection

The survey was disseminated to SIFAC members through official mailing list communications. Fifty clinical pharmacists from the SIFAC network expressed interest in participating; 33 ultimately provided data.

Eligible participants were adults aged 18 years or older attending the pharmacy for any reason during the data collection period. Individuals were excluded if they did not consent to the processing of their personal data for research purposes or were unable to complete the questionnaire independently due to cognitive or language barriers. Each pharmacist was invited to administer the questionnaire to approximately 10 pharmacy users.

The sample calculation is based on the following assumptions: considering that individuals who went to the pharmacy for any reason were invited to participate in the survey and that, according to a 2025 report produced by Federfarma (the Italian Federation of Community Pharmacies) [21], 4 million Italians have access to pharmacy services on a daily basis, we used the Raosoft Sample Size Calculator (Raosoft Inc., Seattle, WA, USA) [22] to calculate an estimate of a proportion in a large population. Considering a confidence level of 95% and a margin of error of 5%, a sample of 385 subjects was taken into account. In order to compensate for any exclusions from the analysis or ineligible questionnaires, we decided to increase the sample size by 5%, aiming for a final sample of 404 interviewed subjects. This calculation was intended to provide an adequate number of observations for the planned descriptive analysis.

To enhance geographic diversity, participants were recruited through community pharmacies located across different Italian regions and in both urban and rural areas.

Participants were enrolled using a convenience sampling approach; therefore, selection bias cannot be excluded.

To reduce potential time-of-day selection bias and increase the likelihood of including individuals with different daily routines and age profiles, questionnaire administration was distributed across three daily time windows: morning (08:00 to 12:00), afternoon (12:00 to 16:00), and evening (16:00 to 20:00).

Prior to administration, each participant received a brief written description of the aims of the survey and the procedures for participation. The questionnaire was administered digitally via Google Forms between 20 October and 10 December 2025.

### 2.3. Statistical Analysis

Data were analyzed using descriptive statistics, including absolute and relative frequencies, means, and standard deviations. For multiple-response items, results were reported both as the percentage of respondents selecting each option and as the percentage of total responses. Where relevant, data were stratified by sex group. Owing to the descriptive objectives of the study, no hypothesis testing or multivariable analyses were performed. All analyses were performed using Microsoft Excel 2024 (Microsoft Corporation, Redmond, WA, USA).

### 2.4. Ethical Considerations

Participation was anonymous and voluntary. Each participant provided consent to the processing of their personal data prior to completing the questionnaire, in accordance with Italian privacy legislation (Legislative Decree 196/2003, as amended by GDPR Regulation EU 2016/679) and in compliance with the ethical standards of the Declaration of Helsinki and its subsequent amendments. No sensitive personal data beyond those strictly necessary for the analysis were collected.

## 3. Results

### 3.1. Sample Characteristics

A total of 406 adults were enrolled across all four Italian geographic macroareas, with representation from 16 regions; the full regional distribution is provided in Supplementary Table S2. Two-thirds of respondents were female (*n* = 271, 66.8%), and the working-age group (36 to 60 years) accounted for the majority of the sample (*n* = 213, 52.5%). Full sociodemographic characteristics are summarized in Table 1.

### 3.2. Perception and Definition of Abdominal Pain

**Figure 1:**
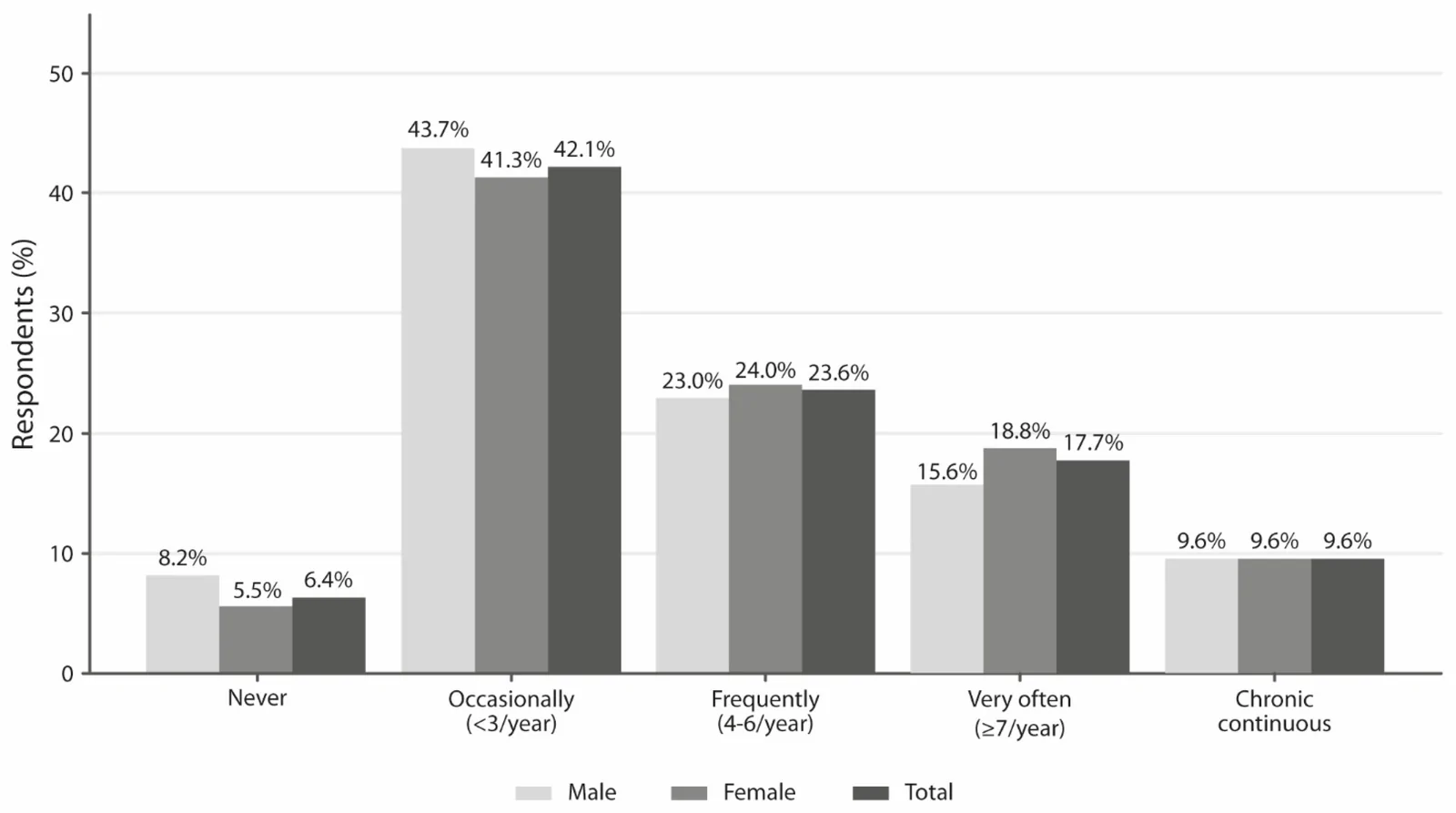
Frequency of abdominal pain episodes in the previous 12 months, stratified by sex (*N* = 406). Values are expressed as percentages.

Over 93% (*n* = 378) of respondents reported at least one episode of abdominal pain in the preceding 12 months. The most frequently reported pattern was occasional (fewer than three episodes per year; *n* = 171, 42.1%), followed by frequent (four to six per year; *n* = 96, 23.7%), very often (seven or more per year; *n* = 72, 17.7%), and chronic or continuous (*n* = 39, 9.6%). Together, more than one in four respondents described a pain pattern of at least seven episodes per year or chronic/continuous occurrence. The distribution of episode frequency appeared broadly similar between sexes (Figure 1).

When asked to classify the etiology, among the subjects who reported at least one episode of stomachache, 83.7% (*n* = 340) attributed their pain to gastrointestinal causes, while 9.4% (*n* = 38) associated it with menstrual pain. The most commonly reported localization was the lower abdomen (*n* = 166, 43.9%), followed by the upper abdomen (*n* = 61, 16.1%), the central abdominal area (*n* = 61, 16.1%), the pelvic area (*n* = 31, 8.2%), and diffuse pain across the entire abdomen (*n* = 48, 12.7%). Only 2.9% (*n* = 11) could not identify a specific area.

The most frequent symptom was cramping pain (*n* = 132, 34.9%), followed by bloating (*n* = 50, 13.2%), diarrhea (*n* = 43, 11.4%), gastric burning or acidity (*n* = 38, 10.1%), and menstrual pain (*n* = 38, 10.1% of all responses, exclusively in women).

**Figure 2:**
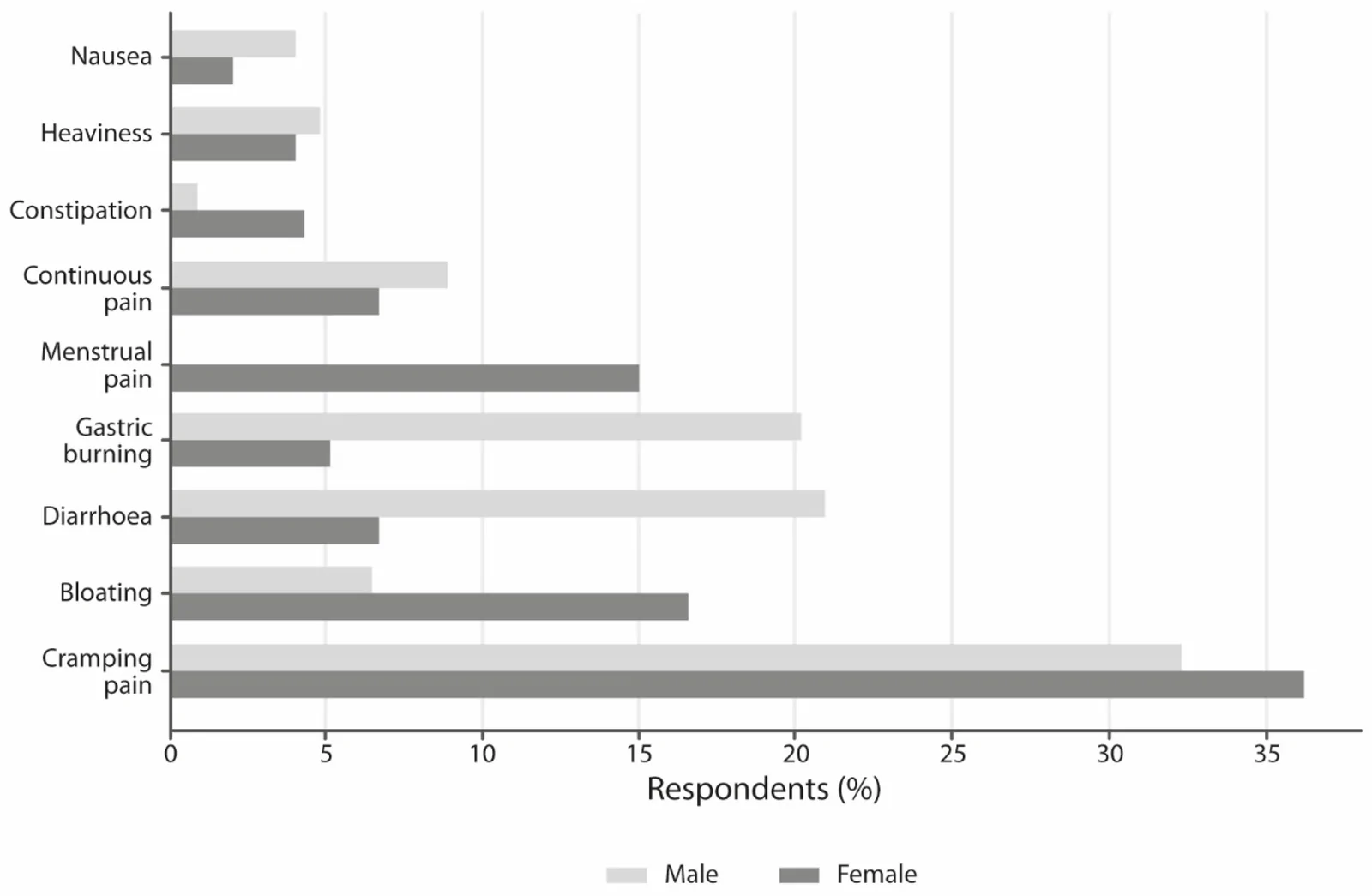
Symptoms associated with abdominal pain, stratified by sex (*N* = 378). Values are expressed as percentages of respondents.

Descriptive differences in symptom reporting were observed between sexes (Figure 2). Among male respondents, diarrhea was experienced by 21.0% (*n* = 26) compared with 6.7% (*n* = 17) of females, while gastric burning affected 20.2% (*n* = 25) and 5.1% (*n* = 13), respectively. Conversely, bloating was reported by 16.5% (*n* = 42) of females and 6.5% (*n* = 8) of males. Menstrual pain occurred in 15.0% (*n* = 38) of the surveyed women.

**Figure 3:**
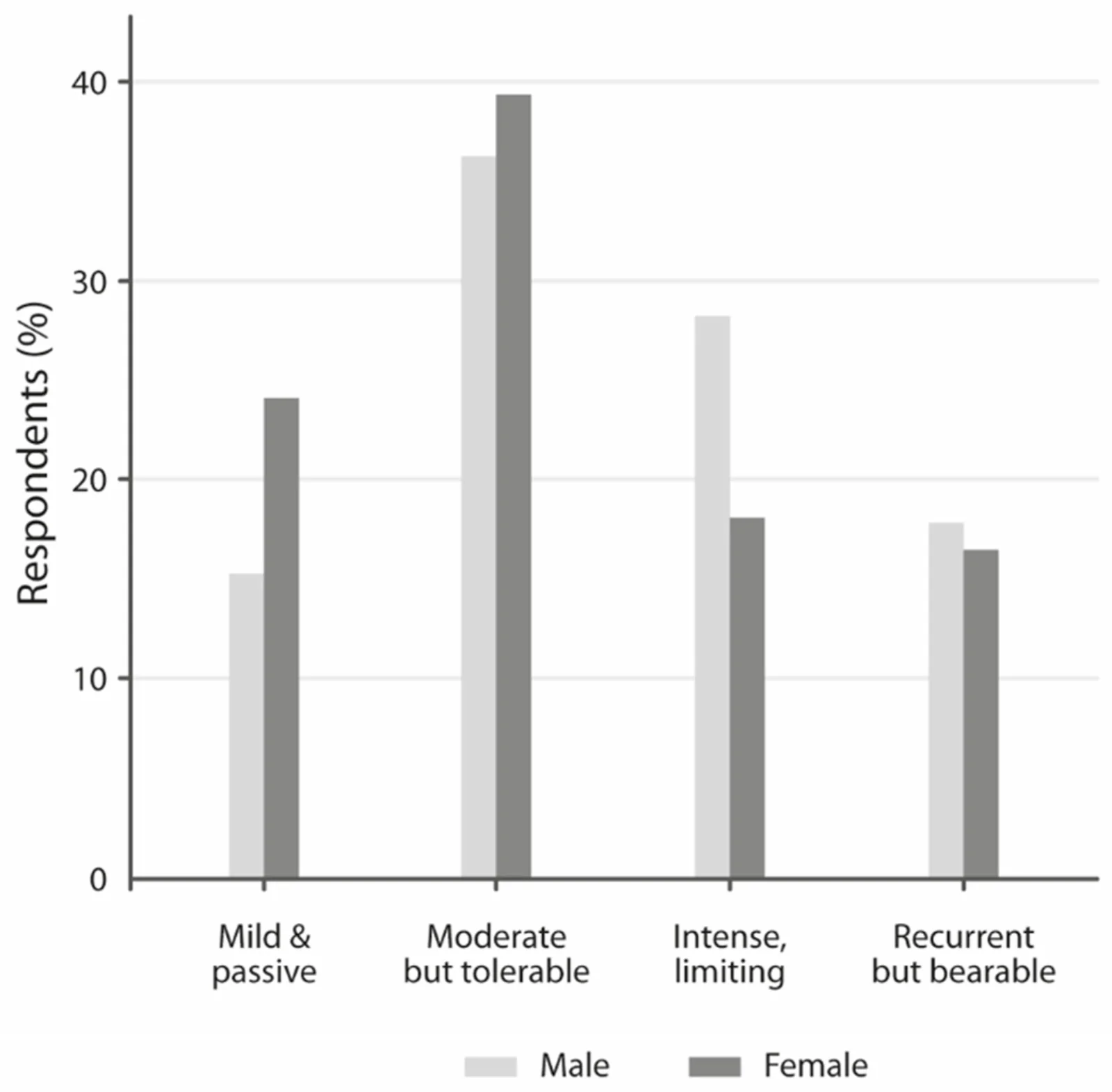
Perceived intensity, stratified by sex (*N* = 378). Values are expressed as percentages of respondents.

When asked about perceived intensity, the majority of respondents did not regard their pain as trivial: 38.4% (*n* = 145) described it as moderate but tolerable, 21.4% (*n* = 81) as intense and limiting daily activities, and 16.9% (*n* = 64) as recurrent but bearable. Only 21.2% (*n* = 80) considered it mild and transient. Perceived intensity by sex is shown in Figure 3.

Dietary problems, including heavy meals and food intolerances, were the most frequently attributed cause (*n* = 168, 44.4% of respondents), followed by emotional stress (*n* = 128, 33.9%), chronic gastrointestinal conditions such as irritable bowel syndrome or gastritis (*n* = 82, 21.7%), and intestinal infections (*n* = 69, 18.3%). All these causes, which are self-reported, were perceived by the respondents as triggering factors for abdominal pain.

Among women, the menstrual cycle was relevant for 24.4% (*n* = 62). A minority (*n* = 29, 7.7%) could not identify any cause. More than half of respondents (*n* = 202, 53.4%) attributed their pain to a single cause; the remainder indicated two contributing factors.

### 3.3. Healthcare-Seeking Behaviors and Needs

The predominant expressed need when experiencing abdominal pain was rapid pharmacological relief of the main symptom (*n* = 186, 49.2%), although 30.4% (*n* = 115) sought relief specifically without medication. Identifying the underlying cause was a priority for 28.3% (*n* = 107) and preventing recurrence for 19.8% (*n* = 75). A smaller proportion (*n* = 32, 8.7%) sought reassurance and education from a healthcare professional as their primary need. When stratified by sex, male respondents more commonly prioritized pharmacological relief (*n* = 72, 58.1% versus *n* = 114, 44.9% of females), while females more frequently sought non-pharmacological approaches (*n* = 84, 33.1% versus *n* = 31, 25.0% of males).

The community pharmacist was identified as the preferred professional across a range of clinical situations, from mild-to-moderate to high-intensity episodes, while the physician was consulted predominantly when symptoms were intense and accompanied by alarm signs or in the presence of a first-time or pre-existing gastrointestinal condition. Notably, 11.9% reported never consulting a physician (Table 2).

### 3.4. Pharmacy Services and Additional Support Needs

Participants were asked to rate the importance of five community pharmacy services in the context of abdominal pain using a four-point scale. The five services evaluated were identification of situations requiring medical referral, medication counselling, advice on prevention and lifestyle, information on symptoms, and advice on natural products and supplements. The combination of “fairly important” and “very important” responses exceeded 80% for all five services, indicating a broadly positive perception of the pharmacist’s role across each domain.

Identification of situations requiring medical referral was the most frequent item rated as “very important” (*n* = 230, 60.9%), followed by medication counselling (*n* = 196, 51.9%), advice on natural products and supplements (*n* = 162, 42.9%), advice on prevention and lifestyle (*n* = 165, 43.7%), and information on symptoms (*n* = 160, 42.3%).

When asked about additional support they would find useful during a pharmacy consultation, 58.5% (*n* = 221) of respondents requested clear and personalized information on therapies and prevention, 42.6% (*n* = 161) wanted better explanations of possible causes, 29.1% (*n* = 110) sought guidance on when to consult a physician, and 17.7% (*n* = 67) asked for more time and attentive listening during the consultation.

### 3.5. Self-Care Behaviors

Among the 378 respondents with at least one episode, 89.4% (*n* = 338) reported using one or more products for self-medication; only 10.6% (*n* = 40) did not use any. Among self-medication users, 51.3% (*n* = 194) took products only when pain was intense or limiting daily activities, 20.4% (*n* = 77) situationally (when facing imminent commitments or when away from home), and 17.7% (*n* = 67) always, regardless of intensity. Polytherapy was the norm: two-thirds (*n* = 225, 66.6%) combined two product categories, and nearly one-third (*n* = 107, 31.7%) used three.

The most frequently used categories were probiotics and intestinal flora products (*n* = 194, 57.4%), antispasmodics (*n* = 148, 43.8%), and antacids and gastric acid suppressants (*n* = 110, 32.5). Use of analgesics and anti-inflammatory agents was reported by 36.2% (*n* = 83) of females and 13.8% (*n* = 15) of males (Table 3).

Healthcare professionals (pharmacists or physicians) were the primary information source before product use for 90.2% (*n* = 305) of respondents. Digital sources (specialized websites, social media, and search engines) were consulted by 23.4% (*n* = 79), and informal advice from family or friends by 22.8% (*n* = 77).

Despite high self-medication prevalence, confidence in autonomous management without immediate professional support was only moderate. Only 44.7% of self-medication users rated themselves very or completely confident in managing abdominal pain autonomously, while the remaining 55.3% reported low or very low confidence. Insecurity was noted in 64.2% of males (*n* = 70) and 51.1% of females (*n* = 117).

## 4. Discussion

This survey provides, to our knowledge, the first systematic account of how adults attending Italian community pharmacies perceive, define, and self-manage abdominal pain. Overall, the findings highlight the heterogeneous nature of abdominal pain, the relevant role of the community pharmacist in its initial management, and the frequent use of self-medication despite limited confidence in autonomous symptom management. These findings have implications for symptom assessment, counselling, and the identification of situations requiring medical evaluation.

### 4.1. Semantic Complexity and Its Implications for Counselling

The heterogeneous symptom profiles reported in this survey confirm that the term “abdominal pain” functions as an umbrella designation for a wide range of clinically distinct conditions. Lower abdominal cramping, epigastric burning, generalized bloating, diarrhea, and menstrual pain were all described using the same lay expression, yet each corresponds to a different clinical picture and a different management pathway. In the absence of structured symptom assessment, presentations requiring medical referral may be indistinguishable from those amenable to self-care. This challenge has been documented in different research contexts.

A survey-based study conducted in Italian community pharmacies found that individuals presenting with upper gastrointestinal complaints described a wide and heterogeneous range of symptom profiles, with epigastric burning (31.8%), acid regurgitation (14.6%), and postprandial fullness (12.0%) being the most frequently reported. The study also showed considerable variability in treatment patterns, with proton pump inhibitors (35.6%), antacids (17.5%), and alginate-based products (17.2%) being the most commonly used [14]. These findings further support the need for a symptom-oriented and individualized approach to the management of upper gastrointestinal complaints. Studies evaluating pharmacist responses to standardized patient scenarios have further shown that history-taking in these encounters is often incomplete: questions about symptom duration are asked in a minority of interactions, alarm features and prior medical history are frequently omitted, and referral criteria are applied inconsistently [16,17,23,24]. Together, these findings suggest that the gap between recommended and actual triage practice in community pharmacy is not a marginal or context-specific problem but a recurrent pattern documented across different countries and methodological approaches.

The observation that more than one in four respondents reported a pattern of frequent or chronic pain episodes warrants consideration. Recurrent abdominal pain is recognized as a source of functional impairment and reduced health-related quality of life across the general population, with functional gastrointestinal disorders affecting more than 40% of adults worldwide [3]. In the working-age group, the occupational consequences can be substantial: studies on functional gastrointestinal conditions characterized by abdominal pain report that symptoms affect work productivity for multiple days each month and lead to recurrent absenteeism [25].

This dimension of the complaint is rarely captured in single-episode study designs; patient-reported surveys are better placed to reveal it. The perceived intensity data reinforce this picture: a substantial proportion of respondents described their pain as intense or limiting daily activities, a finding difficult to reconcile with a view of abdominal pain as a uniformly minor ailment.

Descriptive differences in symptom reporting were observed between male and female respondents. In this survey, males more commonly associated their pain with diarrhea and gastric burning, while females more frequently reported bloating, cramping, and menstrual pain. Although no formal statistical comparisons were performed, these descriptive patterns may be considered alongside previous evidence on sex-related differences in functional gastrointestinal symptom profiles, whereby bloating and constipation are more prevalent among women, and diarrhea is more common among men, with female symptoms further modulated by hormonal factors across the menstrual cycle [26].

These observations, together with previous evidence on sex-related variation in gastrointestinal symptoms, support the importance of structured, patient-centered symptom assessments that take individual clinical characteristics into account.

Structured, patient-centered history-taking is therefore a prerequisite for safe pharmaceutical care in this context, not a desirable enhancement of it. Evidence from Spanish community pharmacies supports the value of structured approaches. In a pre–post study, a validated algorithm for upper gastrointestinal symptoms was associated with significant improvements in Gastroesophageal Reflux Disease Impact Scale (GIS) scores in patients with retrosternal (mean change 1.09, SD 4.28; *p* < 0.001) and overlapping symptoms (mean change 3.18, SD 6.01; *p* < 0.001), with over 99.2% of patients reporting satisfaction with pharmaceutical care received [27]. Developing and validating an analogous decision-support tool for the broader and more heterogeneous spectrum of abdominal presentations documented in this survey would represent a concrete and evidence-informed step towards improving triage practice in Italian community pharmacies.

### 4.2. The Pharmacist as First-Line Healthcare Professional

The consultation patterns observed in this survey suggest that pharmacy customers perceive the pharmacist as an important professional within the initial management pathway for abdominal pain, particularly for mild-to-moderate episodes and for high-intensity episodes without alarm signs, whereas physician consultation was more commonly associated with high-intensity episodes accompanied by alarm signs. Together with the high importance attributed to pharmacist-provided counselling and guidance on medical referral, these findings place the community pharmacist at the interface between self-care and medical evaluation. Rather than replacing medical assessment, the pharmacist may support symptom management and help identify situations in which self-medication is no longer appropriate through effective assessment, counselling, and timely referral.

This interpretation is consistent with international evidence from different healthcare systems, in which community pharmacists are perceived as accessible, trustworthy, and competent sources of advice for symptomatic complaints [19,28,29,30,31,32,33]. In Italy, this role is sustained by standard university curricula, which align with European directives by integrating clinical pharmacy into the core degree for pharmacists [34].

A systematic review of patient and public perspectives on community pharmacies in the United Kingdom identified accessibility, perceived benefits, and recognition of pharmacists’ clinical skills as important factors influencing the uptake of community pharmacy services [11]. The review indicated that patients and the public generally perceived pharmacy services as beneficial, while successful integration of extended pharmacy services was considered dependent, among other factors, on patients’ and physicians’ recognition of pharmacists’ clinical skills. Notably, positive perceptions of pharmacists appear to extend beyond the traditional pharmacy setting. Qualitative research conducted in the United Kingdom found that patients valued accessibility, attentive listening, positive interactions, and the opportunity to receive immediate feedback when pharmacists were integrated into general practice teams [35]. These findings suggest that patient confidence in pharmacists may be supported not only by the setting in which care is delivered but also by accessibility, professional competence, and the quality of the pharmacist–patient relationship.

### 4.3. Self-Care Behavior and Perceived Competence in Autonomous Management

The observation that the majority of respondents self-managed their abdominal pain while simultaneously reporting low or very low confidence in doing so has parallels in the broader literature on self-management of pain-related conditions. A qualitative study found that patients managing chronic pain medications frequently navigated complex decisions about adverse effects with limited professional input and that dilemmas and discrepancies between patients and pharmacists in prioritizing pain relief versus safety were common [36]. This is compounded in the pharmacy context by evidence that pharmacists themselves often feel insufficiently equipped to provide structured guidance on pain-related self-medication [37] and that inadequate addressing of patient concerns during consultations tends to reduce engagement with professional advice over time [18].

The distribution of product categories closely mirrors the symptom profiles reported in the survey: probiotics align with the frequency of gastrointestinal attributions; antispasmodics correspond to the dominance of cramping; and the higher use of antacids among males aligns with their greater prevalence of gastric burning. This correspondence suggests that product choices may reflect how individuals interpret their symptoms, highlighting the importance of pharmacist counselling in ensuring that self-medication decisions are based on appropriate symptom assessment and treatment selection. The frequent combination of multiple product categories raises further questions about therapeutic rationale, appropriate duration, and escalation criteria that are unlikely to be addressed without a structured consultation.

The higher reported use of analgesics and anti-inflammatory agents among female respondents may partly be related to the management of dysmenorrhea. Indeed, a large nationwide study reported that dysmenorrhea affects up to 85% of women and often remains unspoken because of limited communication with both family members and healthcare professionals [38]. Moreover, an Italian cross-sectional study of 290 women with pelvic pain found that, although 72.2% had previously consulted a physician, only 28% had received a diagnosis. The mean delay before seeking medical consultation was 2.5 ± 4.3 years, and only 40.2% received their first treatment from a physician, whereas the remainder relied on pharmacist recommendations or self-medication [39]. These findings highlight the need for appropriate patient counselling, greater awareness of menstrual and pelvic pain, and timely medical evaluation.

These considerations converge on a common need: structured pharmacist counselling that goes beyond product recommendation to address the rationale for treatment, the boundaries of self-management, and the criteria for seeking medical assessment. The unmet needs identified in this survey—personalized information on therapies and prevention, better explanations of possible causes, guidance on when to consult a physician, and more time for attentive listening—suggest that patient needs may extend beyond simple access to the pharmacist and may require greater depth and personalization of the clinical interaction. Individuals value pharmacists who provide personalized, non-judgmental advice and clear explanations over those who prioritize transaction speed or product availability [11], and in the context of pain management, patients have similarly been found to value pharmacists who acknowledge the complexity of their condition rather than limiting the interaction to product dispensing [40]. The high frequency of responses identifying referral as a key function indicates that individuals expect the pharmacist to exercise a reliable triage function, not merely a dispensing one.

Four service development priorities follow from these findings. First, pharmacist training should include structured abdominal symptom assessment, covering localization, alarm features, sex-specific presentations, and referral criteria. The need for such training is supported by evidence that pharmacists themselves perceive pain-related presentations as among the most challenging to manage in the community pharmacy setting: a qualitative study conducted in Ontario found that pharmacists, despite being knowledgeable and empathetic, reported gaps in training, difficulties in monitoring, and systemic barriers as significant impediments to providing effective care to patients with chronic pain [37].

Second, validated decision-support algorithms for abdominal pain triage would reduce the variability in pharmacist assessment and referral practice documented across different national contexts, including incomplete symptom characterization and inconsistent application of referral criteria in studies evaluating pharmacist responses to standardized patient scenarios [16,17,23,24]. The feasibility and impact of this approach have been demonstrated in a cluster randomized trial conducted in Spanish community pharmacies. In this study, a standardized Minor Ailment Service (MAS) incorporating structured protocols for gastrointestinal disturbances (including diarrhea, flatulence, heartburn, and vomiting), pain, and respiratory symptoms significantly improved the appropriateness of medical referral compared with usual care. The trial found that patients receiving protocol-guided MAS had more than twice the odds of an appropriate medical referral compared with those receiving usual care (OR 2.34, 95% CI 1.15–4.79) [41].

Third, accessible patient education materials covering common causes of abdominal pain, appropriate use of over-the-counter product categories, and the signs that should prompt medical consultation would directly address the information gap identified by the majority of respondents. In a randomized controlled trial, pharmacist-led education, lifestyle, and dietary intervention resulted in greater QoL improvement after 8 weeks compared with standard medical therapy alone (20.1 ± 12.1 vs. 13.2 ± 13.4), with multivariate analysis confirming a significant positive effect of pharmacist intervention (B = 5.9; 95% CI, 2.4–9.4; *p* = 0.001) [42]. These findings provide evidence that pharmacist-led educational interventions can improve patient outcomes in gastrointestinal conditions.

Fourth, digital resources targeting younger adults offer an opportunity to extend pharmacist-led guidance beyond the physical consultation encounter. Young adults rely predominantly on digital platforms, particularly general search engines and social media, as their primary entry points for health information, with platform design features such as interactivity, visual engagement, and accessibility playing a key role in shaping trust and content engagement [43]; pharmacist-endorsed digital content addressing common abdominal complaints could intercept these information-seeking behaviors and provide reliable, professionally grounded guidance at the point of need.

### 4.4. Limitations

This study has several limitations that should be considered when interpreting its findings. The cross-sectional design precludes causal inference and does not allow tracking of how perceptions or behaviors evolve over time. The sample size was considered adequate for the planned descriptive analysis and was exceeded by the final sample of 406 respondents. However, because participants were recruited through convenience sampling, statistical representativeness of the Italian general population cannot be assumed. Individuals who regularly access community pharmacy services may be more likely to be included, potentially limiting the generalizability of the findings. In addition, recruitment within community pharmacies may have influenced participants’ perspectives on pharmacy-based care. Recruitment was distributed across different daily time windows (morning, afternoon, and evening) to increase the likelihood of including individuals with different daily routines and age profiles. Nevertheless, the possibility of selection bias should be considered when interpreting the results.

The predominantly female sample reflects the well-documented pattern of greater female utilization of community pharmacy services [14,19], also reported in surveys conducted by patients or trade associations [44]. However, this imbalance may introduce gender bias in the estimates reported for the overall sample. Self-reported data carry inherent recall and social desirability limitations and may underestimate product use that respondents consider inappropriate. The descriptive analytical approach does not permit multivariate exploration of associations between variables such as self-management confidence, symptom severity, and demographic characteristics.

As a methodological strength, the involvement of SIFAC-affiliated community pharmacists with advanced clinical training may have contributed to standardized questionnaire administration and high-quality data collection.

## 5. Conclusions

This survey provides, to our knowledge, the first systematic description of how adults attending Italian community pharmacies perceive, define, and self-manage abdominal pain. The data highlight the heterogeneous nature of the symptom and suggest that pharmacy customers perceive the pharmacist as an important point of contact in its initial management, whereas physician consultation is more commonly sought in specific clinical situations, particularly when abdominal pain is accompanied by alarm signs. The widespread use of self-medication, together with the limited confidence reported by many respondents in autonomous symptom management, highlights the need for support that extends beyond product selection.

The findings support the potential value of structured symptom assessment, personalized counselling, and clear guidance on when medical evaluation is warranted. They also provide a rationale for further developing and evaluating pharmacist training, decision-support tools, and patient education materials tailored to abdominal pain presentations.

Future research should build on these findings using larger and multivariate analytical methods to identify the determinants of self-management confidence and healthcare-seeking behavior. Randomized evaluations of structured counselling interventions, conducted in real pharmacy settings and measuring outcomes such as triage accuracy, appropriate referral, and self-management confidence, represent a particularly relevant next step.

## Figures and Tables

**Table 1:** Sociodemographic characteristics of the study sample (*N* = 406).

Characteristic	Total *N* (%)
Sex	406 (100)
- Female	271 (66.8)
- Male	135 (33.2)
Age, years (SD)	
- Mean	44.3 (14.6)
Age group	
- Young adults (18 to 35)	130 (32.0)
- Adults (36 to 60)	213 (52.5)
- Late adults (61 to 74)	50 (12.3)
- Elderly (75 or over)	13 (3.2)
Geographic area	
- North	139 (34.2)
- Centre	63 (15.5)
- South	95 (23.4)
- Islands	109 (26.9)

**Table 2:** Clinical situations prompting consultation with the pharmacist versus the physician (*N* = 378).

Clinical Situation	Pharmacist *N* (%)	Physician *N* (%)
Mild or moderate intensity	113 (29.9)	17 (4.5)
High intensity without alarm signs	152 (40.2)	–
High intensity with alarm signs	–	202 (53.4)
Recurrent episode with known pattern	69 (18.3)	24 (6.4)
First-time episode	44 (11.6)	61 (16.1)
Pre-existing gastrointestinal conditions	–	29 (7.7)
Never consults this professional	–	45 (11.9)

(–) indicates that the corresponding clinical situation was not included as a response option for that healthcare professional.

**Table 3:** Therapeutic product categories used for abdominal pain self-medication, stratified by sex (*N* = 338 self-medication users; *N* = 109 male self-medication users; *N* = 229 female self-medication users; multiple responses allowed, maximum 3).

Product Category	Total *N* (%)	Male *N* (%)	Female *N* (%)
Probiotics and intestinal flora products	194 (57.4)	62 (56.9)	132 (57.6)
Antispasmodics	148 (43.8)	51 (46.8)	97 (42.4)
Antacids and gastric acid suppressants	110 (32.5)	50 (45.9)	60 (26.2)
Analgesics and anti-inflammatory agents	98 (29.0)	15 (13.8)	83 (36.2)
Antidiarrheals	74 (21.9)	35 (32.1)	39 (17.0)
Laxatives	28 (8.3)	4 (3.7)	24 (10.5)

## Data Availability

The data presented in this study are available upon reasonable request to the corresponding author due to confidentiality restrictions.

## Supplementary Materials

The following supporting information can be downloaded at https://www.mdpi.com/article/10.3390/pharmacy14060133/s1, Table S1: Full questionnaire; Table S2: Regional distribution of participants in Italy; File S1: STROBE Statement—Checklist of items that should be included in reports of cross-sectional studies.
