# Supplementary material for: Individual Perceptions and Self-Care Practices for Abdominal Pain: A Cross-Sectional Survey Conducted in Italian Community Pharmacies
Source: Pharmacy (Basel). 2026 Sep 14;14(6):133. doi: 10.3390/pharmacy14060133 (PMC13610268; doi:10.3390/pharmacy14060133)
Supplement: Supplementary file 1 [file pharmacy-14-00133-s001.zip › Table S1.pdf]

TABLE S 1: Full questionnaire, Section A through D

QUESTIONNAIRE

SECTION A – GENERAL INFORMATION (CONSUMER)

Q1. Region

____

Q2. Province

____

Q3. Age

____

Q4. Gender

____

SECTION B – CONSUMER PERCEPTION AND DEFINITION OF "ABDOMINAL PAIN (MAL DI PANCIA)"

Q5. In the last 12 months, how often have you experienced episodes of "abdominal pain" (mal di pancia)? *(Single answer)*

□ Never *(end questionnaire)*

□ Sometimes (fewer than 3 episodes in the last 12 months)

□ Frequently (4–6 episodes in the last 12 months)

□ Very often (7 or more episodes in the last 12 months)

□ Always or almost always (episodes lasting more than a few consecutive days / chronic condition)

□ I don't remember *(end questionnaire)*

Q6. Thinking about the last time you had abdominal pain, when you use the term "abdominal pain" (mal di pancia), what location does it refer to? *(Single answer)*

□ Upper abdomen (stomach area)

□ Middle abdomen (above the navel)

□ Lower abdomen (lower belly area)

□ Pelvic area (uterine area)

□ Entire abdomen / any abdominal area

□ I don’t have a precise location in mind

Q7. Thinking about the last time you had abdominal pain, which of the following signs or symptoms do you mainly associate with the term “abdominal pain” (mal di pancia)? *(Single answer)*

□ Continuous pain

□ Pain in episodes/waves (cramp-like pain)

□ Heartburn / stomach acidity

□ Bloating

□ Feeling of heaviness

□ Nausea

□ Feeling of a “blocked stomach”

□ Diarrhoea

□ Constipation

□ Menstrual pain

□ I don’t know / I wouldn’t know how to define

□ Other ______________________

Q8. Thinking about the last time you had abdominal pain, when you use the term “abdominal pain” (mal di pancia), you mean it as… *(Single answer)*

□ A mild and temporary problem

□ A moderate but tolerable problem

□ A severe problem that limits activities

□ A manageable problem but one that occurs frequently again

□ I don’t know / I wouldn’t know how to define

Q9. Thinking about the last time you had abdominal pain, in your opinion, what was it mainly caused by? *(You may indicate 2 answers)*

□ Food-related issues (large meals, intolerances)

□ Emotions or stress

□ Infections or intestinal viruses

□ Menstrual period

□ Chronic conditions (irritable bowel syndrome, gastritis, etc.)

□ I don’t know / I wouldn’t know how to define

□ Other ___________

SECTION C – CHARACTERISATION OF THE PROBLEM, CONSUMER NEEDS AND HEALTHCARE PROFESSIONAL NEEDS

Q10. When you have an episode of “abdominal pain” (mal di pancia), what is your main need? *(You may indicate 2 answers)*

□ To quickly reduce the main symptom by using medicines

□ To quickly reduce the main symptom without using medicines

□ To identify the cause of the problem

□ To prevent it from happening again

□ To receive reassurance/education from a healthcare professional

□ Other________________

Q11. In which of the following situations do you tend to contact the pharmacist for “abdominal pain” (mal di pancia)? *(Single answer)*

□ When the problem is of mild/intermediate intensity

□ When the problem is of high intensity

□ When a new type of abdominal pain appears for the first time

□ When the problem is recurring but already known

Q12. In which of the following situations do you tend to contact a doctor for “abdominal pain” (mal di pancia)? *(Single answer)*

□ When the problem is of mild/intermediate intensity

□ When the problem is of high intensity and is associated with other symptoms

□ When a new type of abdominal pain appears for the first time

□ When the problem is recurring but already known

□ In case of previous or chronic gastrointestinal conditions

□ I do not contact a doctor

Q13. How important do you think it is to receive the following services from the pharmacist for managing “abdominal pain” (mal di pancia)?

For each area, indicate a value from **1 to 4**, where:

1 = Not at all important; 2 = Slightly important; 3 = Fairly important; 4 = Very important.

a) Advice on medicines 1 2 3 4

b) Advice on natural products or supplements 1 2 3 4

c) Identifying situations that should be referred to the doctor 1 2 3 4

d) Information on symptoms and manifestations of “abdominal pain” 1 2 3 4

e) Advice on prevention, diet, and lifestyle 1 2 3 4

Q14. If you could receive additional support from the pharmacist on the topic of “abdominal pain” (mal di pancia), which would you prefer? *(You may indicate 2 options)*

□ More listening and time dedicated to the consultation

□ Simple and clear information on possible causes

□ Simple and clear information on treatments and prevention

□ Guidance on when to consult a doctor

□ Informational materials and educational brochures

□ Other____________

SECTION D – SELF-CARE AND PROBLEM MANAGEMENT

Q15. When you have abdominal pain, what do you do? *(Single answer)*

□ I always take a product to manage it

□ I take a product when the abdominal pain is intense and limits my normal activities

□ I take a product only when I need to deal with an upcoming commitment or I'm out of the house

□ I never take products *(end questionnaire)*

Q16. What are the main channels you use to look for information before purchasing the product? *(You may indicate 2 options)*

□ I ask people who have already used the product (family, friends, acquaintances)

□ I always consult a healthcare professional (doctor, pharmacist)

□ I consult specialised websites, social networks, or search engines

□ Television

□ Radio

□ Newspapers/magazines

□ Other __________________

Q17. Which of the following categories of products have you used the last time you had abdominal pain? *(Maximum 3 answers)*

□ Painkillers (analgesics/anti-inflammatory drugs)

□ Products for abdominal spasms/cramps (antispasmodics)

□ Products for heartburn (antacids/gastric secretion inhibitors)

□ Products for diarrhoea (anti-diarrhoeal drugs)

□ Products to restore intestinal bacterial flora (probiotics/lactic ferments/prebiotics)

□ Products for constipation (laxatives)

□ I do not use therapies/products for abdominal pain

□ Other __________________

Q18. How safe do you feel/would you feel in managing an episode of abdominal pain (mal di pancia) yourself without immediately consulting a healthcare professional (pharmacist, general practitioner, or specialist)? *(Single answer)*

□ Not at all confident

□ Not very confident

□ Very confident

□ Completely confident
