# Supplementary material for: Individual Perceptions and Self-Care Practices for Abdominal Pain: A Cross-Sectional Survey Conducted in Italian Community Pharmacies
Source: Pharmacy (Basel). 2026 Sep 14;14(6):133. doi: 10.3390/pharmacy14060133 (PMC13610268; doi:10.3390/pharmacy14060133)
Supplement: Supplementary file 1 [file pharmacy-14-00133-s001.zip › Table S2.pdf]

TABLE S2 : Regional distribution of partecipants in Italy

	**Total** ***N (%)***
Region	406 (100)
- Abruzzo	14 (3.45)
- Calabria	14 (3.45)
- Campania	39 (9.61)
- Emilia-Romagna	6 (1.48)
- Friuli-Venezia Giulia	25 (6.16)
- Lazio	27 (6.65)
- Liguria	15 (3.69)
- Lombardia	63 (15.52)
- Marche	21 (5.17)
- Molise	3 (0.74)
- Puglia	25 (6.16)
- Sardegna	63 (15.52)
- Sicilia	46 (11.33)
- Toscana	14 (3.45)
- Umbria	1 (0.25)
- Veneto	30 (7.39)
Geografical Area	406 (100)
- North	139 (34.23)
- Center	63 (15.52)
- South	95 (23.40)
- Islands	109 (26.85)
